# Task Relevance Over Emotional Factors in Older Adults’ Non-Formal Learning

**DOI:** 10.1093/geroni/igaf122.2955

**Published:** 2025-12-31

**Authors:** Yang Wang, Heng Zhao, Huamao Peng

**Affiliations:** Beijing Normal University Institute of Developmental Psychology, Beijing, Beijing, China (People’s Republic); Beijing Normal University Institute of Developmental Psychology, Beijing, Beijing, China (People’s Republic); Beijing Normal University Institute of Developmental Psychology, Beijing, Beijing, China (People’s Republic)

## Abstract

Despite the prevalence of non-formal learning among older adults, research in this area remains limited. Therefore, this study aims to analyze older adults’ non-formal learning processes, with a particular focus on continuance intention of learning, and to compare the distinct effects of task relevance and emotional factors. Experiment 1 was conducted in a laboratory setting, involving 77 young adults (Mage = 22.49, 18-30 years) and 80 older adults (Mage = 68.41, 61-81 years), randomly assigned to three groups: positive emotion, negative emotion, and control, each receiving different manipulations of learning-related emotion. Participants completed both high-relevance and low-relevance learning tasks. Experiment 2 was conducted in a real-world context, involving 44 older adults (Mage = 69.43, 63-78 years) who were randomly assigned to two groups, with one group completing high-relevance courses and the other group completing low-relevance courses during a six-day intensive tracking study. Both experiments measured participants’ continuance intention of learning, learning performance, and subjective mental effort. Additionally, experiment 1 assessed objective mental effort through changes in pupil diameter. The results proved that increased task relevance significantly enhanced older adults’ continuance intention of learning, mental effort, and learning performance in non-formal learning contexts, while its impact on young adults was negligible. Meanwhile, positive emotion was a significant predictor of older adults’ continuance intention of learning, but only under low-task-relevance conditions. Our study confirms the crucial role of task relevance in non-formal learning for older adults, surpassing the impact of emotional factors, providing clear guidance for curriculum design and educational practices.

